# Comparison of the lifetime host-to-tick transmission between two strains of the Lyme disease pathogen *Borrelia afzelii*

**DOI:** 10.1186/s13071-016-1929-z

**Published:** 2016-12-16

**Authors:** Maxime Jacquet, Gabriele Margos, Volker Fingerle, Maarten J. Voordouw

**Affiliations:** 1 Laboratory of Ecology and Evolution of Parasites, Institute of Biology, University of Neuchâtel, Neuchâtel, Switzerland; 2 National Reference Centre for Borrelia, Munich, Oberschleissheim Germany; 3 Bavarian Health and Food Safety Authority, Munich, Oberschleissheim Germany

**Keywords:** *Borrelia afzelii*, Co-feeding transmission, *Ixodes ricinus*, Life-history strategy, Lyme borreliosis, Spirochete, Systemic transmission, Tick-borne pathogen, Vector-borne pathogen

## Abstract

**Background:**

Transmission from the vertebrate host to the arthropod vector is a critical step in the life-cycle of any vector-borne pathogen. How the probability of host-to-vector transmission changes over the duration of the infection is an important predictor of pathogen fitness. The Lyme disease pathogen *Borrelia afzelii* is transmitted by *Ixodes ricinus* ticks and establishes a chronic infection inside rodent reservoir hosts. The present study compares the temporal pattern of host-to-tick transmission between two strains of *B. afzelii*.

**Methods:**

Laboratory mice were experimentally infected via tick bite with one of two strains of *B. afzelii*: A3 and A10. Mice were repeatedly infested with pathogen-free larval *Ixodes ricinus* ticks over a period of 4 months. Engorged larval ticks moulted into nymphal ticks that were tested for infection with *B. afzelii* using qPCR. The proportion of infected nymphs was used to characterize the pattern of host-to-tick transmission over time.

**Results:**

Both strains of *B. afzelii* followed a similar pattern of host-to-tick transmission. Transmission decreased from the acute to the chronic phase of the infection by 16.1 and 29.3% for strains A3 and A10, respectively. Comparison between strains found no evidence of a trade-off in transmission between the acute and chronic phase of infection. Strain A10 had higher lifetime fitness and established a consistently higher spirochete load in nymphal ticks than strain A3.

**Conclusion:**

Quantifying the relationship between host-to-vector transmission and the age of infection in the host is critical for estimating the lifetime fitness of vector-borne pathogens.

**Electronic supplementary material:**

The online version of this article (doi:10.1186/s13071-016-1929-z) contains supplementary material, which is available to authorized users.

## Background

Many vector-borne pathogens establish long-lived chronic infections in their vertebrate reservoir hosts [1–7]. This life-history strategy enhances pathogen fitness because it facilitates transmission to feeding arthropod vectors over a longer period of time. Tick-borne spirochete bacteria that belong to the *Borrelia burgdorferi* (*sensu lato*) (*s.l*.) species complex cause Lyme borreliosis (LB) in humans [7–9]. These tick-borne pathogens establish chronic infections in competent vertebrate reservoir hosts, such as rodents [10–14]. Experimental infection studies with different species of rodents have shown that *B. burgdorferi* (*s.l*.) pathogens can have high host-to-tick transmission to feeding larval ticks over a period of months and even years [10–13]. Theoretical models have shown that the reproductive number (R_0_) of tick-borne pathogens is highly sensitive to the duration of the infectious period and the probability of host-to-tick transmission [15–18].

Host-to-tick transmission success can vary dramatically over the course of the infection. In the first week post-infection (PI), the *Borrelia* pathogen replicates in the host skin at the site of the tick bite before disseminating to multiple organs (~10 days PI) [19, 20]. During this time (~7 days PI), uninfected ticks feeding in close proximity to an infected tick can acquire the spirochete infection via non-systemic or co-feeding transmission [21–26]. Once the *Borrelia* pathogen has established a widespread, multi-organ infection, host-to-tick transmission can occur from the skin anywhere on the vertebrate body and is therefore referred to as systemic transmission [23, 25]. Systemic transmission reaches a maximum (80–100%) between 10 and 40 days depending on the *Borrelia* species and rodent host [10, 12, 27–30]. At the same time, the host develops an IgG antibody response against *Borrelia* (15–30 days PI) [20]. These antibodies reduce the spirochete load in the host tissues [31–34], which reduces the efficacy of systemic transmission [35–37]. During the later chronic phase, the *Borrelia* pathogen employs a variety of strategies to evade the immune system and persist in the vertebrate host [38–40].

Kurtenbach et al. [7] pointed out that many tick-borne pathogens have a ‘boom-and-bust’ life history strategy, where host-to-tick transmission is high during the early acute phase of the infection and lower during the later chronic phase of the infection. A number of studies on *Borrelia* pathogens have shown that host-to-tick transmission peaks during the first four weeks of infection [10, 12, 27], followed by lower transmission after this period, but this is not always the case [11, 28, 30]. Haven et al. [41] pointed out that the relationship between host-to-tick transmission and the age of infection is a critical driver of the epidemiology of LB. They suggested that *Borrelia* pathogens could be divided into inhost persistent strains or rapidly cleared strains [41]. For example, *B. burgdorferi* (*sensu stricto*) (*s.s*.) BL206 is an inhost persistent strain because mouse-to-tick transmission increased from 58.3 to 83.3% from day 10 to day 42 [28]. In contrast, strain B348 is a rapidly cleared strain because transmission decreased from 83.3 to 4.1% over the same time period [28]. A number of studies on the North American LB system of *B. burgdorferi* (*s.s*.) in *I. scapularis* ticks have compared the temporal pattern of host-to-tick transmission between strains [27, 28, 30]. In contrast, no such studies have been performed on European LB pathogens.

*Borrelia afzelii* is the most common cause of LB in Europe. This tick-borne pathogen is transmitted by the tick *Ixodes ricinus* and is specialized on rodent reservoir hosts [42]. We have previously compared host-to-tick transmission between two isolates of *B. afzelii*: E61 and NE4049 [25, 26, 35] during the acute phase of the infection (defined here as 1–35 days PI). These studies found that isolate NE4049 had higher co-feeding transmission (day 2 PI) and systemic transmission (day 34 PI) than isolate E61. However, these studies did not investigate host-to-tick transmission during the chronic phase of the infection (defined here as > 35 days PI). Given the potential for life history trade-offs between the acute and chronic phases of the infection, the purpose of the present study was to test whether isolate NE4049 would maintain its transmission advantage relative to isolate E61 during the chronic phase of the infection. We predicted that isolate E61 would have higher transmission during the chronic phase of the infection than isolate NE4049. This is the first study to compare the temporal pattern of host-to-tick transmission between strains of *B. afzelii*.

## Methods

### Acute phase *versus* chronic phase

In the present study, the acute and chronic phase are defined as ≤ 35 days and > 35 days post-infection (PI). The acute phase contains both co-feeding transmission (2 days PI) and systemic transmission (34 days PI) whereas the chronic phase only has systemic transmission (66, 94, 128 days PI). Our 35-day cut-off between the acute and chronic phase is similar to the 30-day cut-off used by the Centers for Disease Control and Prevention to distinguish between early and later signs and symptoms of LB in humans.

### Strains of *B. afzelii*

*Borrelia afzelii* isolates E61 and NE4049 were used in this study. These isolates have ID numbers 1888 and 1887 in the *Borrelia* multilocus sequence type (MLST) database, respectively. E61 was originally isolated from a human patient in Austria whereas NE4049 was isolated from an *I. ricinus* tick in Neuchâtel, Switzerland. Isolate E61 has sequence type (ST) ST75 and *ospC* major group (oMG) A3 whereas isolate NE4049 has ST679 and oMG A10. For simplicity and as we have done elsewhere, these two isolates will hereafter be referred to as *B. afzelii ospC* strains A3 and A10 [25, 26, 35]. We have previously characterized the co-feeding and systemic transmission phenotypes of these two *ospC* strains over the acute phase of the infection [25, 26, 35].

### Experimental infection of mice with *B. afzelii* via tick bite

We experimentally infected female *Mus musculus* Balb/cByJ mice with either *B. afzelii ospC* strain A3 or strain A10 via nymphal tick bite (total sample size was 41 mice). The details of this infection experiment have been described elsewhere [26, 35]. Briefly, mice were immunized with PBS (control mice) or with one of two recombinant OspC (rOspC) proteins: A3 or A10. All mice were subsequently challenged via tick bite with one of two strains of *B. afzelii*: A3 or A10. All 16 mice in the homologous group (where the *ospC* gene of the challenge strain matched the rOspC immunogen) were protected from infectious challenge. In contrast, 13 of the 15 mice in the heterologous group (where the *ospC* gene of the challenge strain did not match the rOspC immunogen) and the 10 control mice developed a systemic infection following the nymphal challenge. The 18 uninfected mice were excluded from the present study because host-to-tick transmission for these individuals is obviously zero. Of the 23 mice that developed a systemic infection, 10 were infected with strain A3 and 13 were infected with strain A10.

### Measure co-feeding and systemic transmission

To measure co-feeding and systemic transmission, mice were infested with larval *I. ricinus* ticks from our pathogen-free, laboratory colony on five separate occasions at 2, 34, 66, 94 and 128 days after the nymphal challenge. Co-feeding transmission refers to the larval infestation on day 2 PI whereas systemic transmission refers to days 34, 66, 94 and 128 PI. For the first infestation, the co-feeding larvae were placed in a plastic capsule (15 mm in diameter) that was glued to the back of the mouse and that contained the *B. afzelii*-infected challenge nymphs [26]. For each of the remaining four infestations, 50 to 100 larvae were placed on the head of each mouse. Infested mice were placed in individual cages that facilitated the collection of blood-engorged larval ticks. Blood-engorged larvae were placed in individual tubes and were allowed to moult into nymphs [26, 35]. The nymphs were frozen at -20 °C at 4 weeks after moulting into the nymphal stage. For the first infestation and for all subsequent infestations, a maximum of 20 and 10 nymphs were frozen, respectively.

### DNA extraction of nymphal ticks and qPCR to determine *B. afzelii* infection

A total of 1,174 nymphal ticks were processed during the experiment. Total DNA was extracted using a TissueLyser II and DNeasy 96 Blood & Tissue kit well plates (Qiagen, Basel, Switzerland). The DNA extraction protocol was described in a previous study [35]. A quantitative PCR amplifying a fragment of the *flagellin* gene [43] was used to detect and quantify *Borrelia* DNA. The qPCR protocol was described in a previous study [35].

### Statistical analysis

All statistical analyses were done in R version 3.1.0. [44].

#### Effects of the age of infection and strain on the *B. afzelii* infection status of nymphal ticks

Nymphal ticks were considered infected if at least two of the three runs of the qPCR assay tested positive for *B. afzelii*, as described in a previous study [35]. A generalized linear mixed effects (GLME) model with binomial errors was used to model the *B. afzelii* infection status of each nymphal tick as a function of two fixed factors: the age of the infection (2, 34, 66, 94 and 128 days), strain (A3 and A10), and their interaction. Mouse identity was included as a random factor.

#### Effects of the age of infection and strain on the spirochete load of infected nymphal ticks

The spirochete load of each nymphal tick was calculated as the geometric mean of the three replicate runs (negative runs were excluded), as described in a previous study [35]. For the subset of infected nymphs (i.e. uninfected nymphs were excluded), a linear mixed effects (LME) model with normal errors was used to model the log10-transformed spirochete load as a function of the age of the infection, strain, and their interaction. Mouse identity was included as a random factor.

## Results

### Effects of the age of infection and strain on the *B. afzelii* infection status of nymphal ticks

**Fig. 1:**
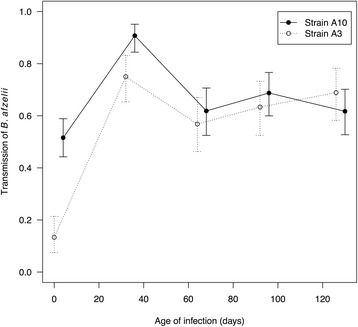
The transmission of *B. afzelii* to *I. ricinus* ticks is shown as a function of the age of infection inside the mouse (2, 34, 66, 94 and 128 days) for each of the two strains, A3 and A10. Transmission data were based on 1,174 *I. ricinus* nymphs that were sampled from 23 mice at all five time points. Shown are the means and the 95% confidence intervals

Strain A10 had higher transmission than strain A3 at all time points except the last one (Fig. 1). Over the duration of systemic transmission (days 34, 66, 94 and 128), the proportions of infected nymphs produced by strains A10 and A3 were 71.0% (358/504) and 66.1% (248/375), respectively. Systemic transmission of *B. afzelii* was highest at the acute phase of the infection (day 34) and then decreased but remained stable over the chronic phase (days 66, 94 and 128; Fig. 1). There was a significant interaction between strain and the age of the infection on transmission (GLME: Δ *χ* ^2^ = 37.326, *df* = 4, *P* < 0.001; Fig. 1). We therefore analysed the effect of the age of infection on the probability of transmission separately for each strain (see below). After removing the interaction from the model, the main effects of strain (GLME: Δ *χ* ^2^ = 9.833, *df* = 1, *P* = 0.002) and the age of infection (GLME: Δ *χ* ^2^ = 128.010, *df* = 4, *P* <0.001) remained highly significant.

For strain A3, the effect of the age of infection was significant (GLME: Δ *χ* ^2^ = 104.72, *df* = 4, *P* < 0.001). Systemic transmission peaked during the acute phase (day 34: 75.0% = 75/100) and was 16.1% lower during the chronic phase (days 66, 94 and 128 combined: 62.9% = 173/275), and this difference was significant (Proportion test: *χ* ^2^ = 4.262, *df* = 1, *P* = 0.039). Co-feeding transmission of strain A3 on day 2 was significantly lower than systemic transmission over all subsequent days (*P* < 0.001). For strain A10, the effect of the age of infection was significant (GLME: Δ *χ* ^2^ = 61.524, *df* = 4, *P* < 0.001). Systemic transmission peaked during the acute phase (day 34: 90.8% = 118/130) and was 29.3% lower during the chronic phase (days 66, 94, and 128 combined: 64.2% = 240/374) and this difference was significant (Proportion test: *χ* ^2^ = 31.887, *df* = 1, *P* < 0.001). Co-feeding transmission of strain A10 on day 2 was significantly lower than systemic transmission over all subsequent days (*P* < 0.001).

### Effects of the age of infection and strain on the spirochete load of infected nymphal ticks

**Fig. 2:**
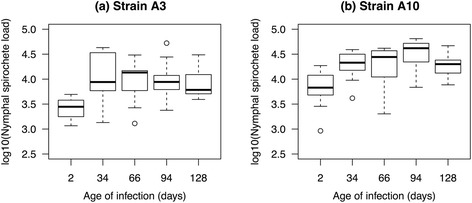
The log10-transformed *B. afzelii* spirochete load of the subset of infected *I. ricinus* nymphal ticks is shown as a function of the age of infection (2, 34, 66, 94 and 128 days) for strain A3 (**a**) and strain A10 (**b**). Spirochete load data were based on 718 *I. ricinus* nymphs infected with *B. afzellii* that were sampled from 23 mice at all five time points. Each data point represents the average nymphal spirochete load for one mouse. Shown are the medians (black line), the 25th and 75th percentiles (edges of the box), the minimum and maximum values (whiskers), and the outliers (circles)

The nymphal spirochete load of strain A10 (~20,000 spirochetes/nymph; Table 1) was consistently higher than that of strain A3 (~10,000 spirochetes/nymph; Table 1, Fig. 2). The interaction between strain and the age of infection on the log10-transformed nymphal spirochete load was not significant (LME: Δ *χ* ^2^ = 3.534, *df* = 4, *P* = 0.473). In the main effects model, the effects of strain (LME: Δ *χ* ^2^ = 10.591, *df* = 1, *P* = 0.001) and age (LME: Δ *χ* ^2^ = 37.085, *df* = 4, *P* < 0.001) were significant. The spirochete load of the nymphs infected via co-feeding transmission on day 2 was significantly lower than that of the nymphs infected via systemic transmission on all subsequent days (*P* < 0.001; Fig. 2). The mean spirochete load was similar between the days where nymphs were infected via systemic transmission (Fig. 2).

**Table 1:** Host-to-tick transmission of *B. afzelii* and the spirochete load of the infected *I. ricinus* nymphs are shown for the ten combinations of strain (A3 or A10) and the age of infection (2, 34, 66, 94 and 128 days). The probability of transmission shows the number of infected nymphs divided by the total number of nymphs analysed, and the corresponding percentage of infected nymphs. The spirochete load presents the mean spirochete load and the 95% confidence interval (CI) for the subset of infected nymphs

Strain	Age of infection (days)	Transmission	Spirochete load	
		Infected nymphs/total nymphs (%)	Mean^a^	95% CI
A3	2	14/105 (13.3)	2,591	1,711–3,924
A3	34	75/100 (75.0)	10,496	8,163–13,496
A3	66	54/95 (56.8)	9,064	7,301–11,251
A3	94	57/90 (63.3)	9,853	7,822–12,411
A3	128	62/90 (68.9)	8,100	6,811–9,633
A10	2	98/190 (51.6)	6,436	5,548–7,465
A10	34	118/130 (90.8)	19,250	17,214–21,528
A10	66	73/118 (61.9)	18,488	15,971–21,402
A10	94	88/128 (68.9)	32,087	28,593–36,009
A10	128	79/128 (61.7)	18,806	17,058–20,735
Total		718/1,174 (61.2)		

^a^For each of the ten combinations of strain and age of infection, the geometric mean spirochete load in the nymphal tick was calculated for each mouse (uninfected nymphs were excluded). For strains A3 and A10, the geometric mean nymphal spirochete load and the 95% confidence interval are based on 10 and 13 mice, respectively

## Discussion

The results of our study were consistent with the boom-and-bust model of host-to-tick transmission proposed by Kurtenbach et al. [7]. For strains A3 and A10, host-to-tick transmission was highest (75.0 and 90.8%, respectively; Table 1) at the end of the acute phase of the infection (day 34 PI), followed by a plateau of lower (but still high) transmission (62.9 and 64.2%, respectively; Table 1) over the chronic phase of the infection (days 66, 94 and 128 days PI). The reduction in the efficiency of systemic transmission between the acute and chronic phase of the infection was 16.1 and 29.3% for strains A3 and A10, respectively. Our results are similar to an earlier experimental infection study of *B. afzelii* in the wood mouse, *Apodemus sylvaticus* [12]. In that study, host-to-tick transmission was very high (100%) during the acute phase (days 18 to 22 PI) followed by a plateau of lower transmission (30–50%) during the chronic phase (weeks 9, 15, 21, 27 and 33 PI) [12]. Others and we have shown that host-to-tick transmission depends on the density of *Borrelia* spirochetes in the host tissues [35–37]. Previous work has shown that *Borrelia*-specific antibodies of the host immune system play a key role in controlling the spirochete load in the host tissues [31–34]. Thus antibodies are the most likely explanation for the observed decrease in host-to-tick transmission from the acute to the chronic phase of the infection [45–47].

Haven et al. [41] suggested that *Borrelia* pathogens could be divided into inhost persistent strains or rapidly cleared strains [41]. In the present study, *B. afzelii* strains A3 and A10 established a chronic infection and are therefore both inhost persistent strains. Strain A10 had consistently higher host-to-tick transmission than strain A3 (except for the last time point) and the temporal pattern of transmission was similar between the two strains (Fig. 1). Thus, comparison of the two strains found no evidence of a transmission trade-off between early and late infection as found in *B. burgdorferi* (*s.s*.) by Haven et al. [41]. Likewise, a previous study comparing six strains of *B. afzelii* in laboratory mice found no evidence of a trade-off between co-feeding transmission and systemic transmission during the acute phase of the infection [25]. In contrast, a study on two North American strains of *B. burgdorferi* (*s.s*.) in the white-footed mouse, *Peromyscus leucopus*, found large differences in the temporal pattern of host-to-tick transmission [27, 28]. For the inhost persistent strain BL206, mouse-to-tick transmission increased from 58.3 to 83.3% from day 10 PI to day 42 PI, whereas for the rapidly cleared strain B348, transmission decreased from 83.3 to 4.1% over the same time period [28]. In summary, our study showed that strain A10 has a higher lifetime transmission success than strain A3.

The relationship between host-to-tick transmission and the age of infection is a critical driver of the epidemiology of LB [41]. The relationship between host-to-tick transmission and the age of infection in the reservoir host is also important for the development of theoretical models. Recently developed next-generation population matrix models typically assume that host-to-tick transmission is constant and high over the duration of the infection [15–17]. We recently used these next-generation matrix methods to estimate the R_0_ value for six different *ospC* strains of *B. afzelii* [25]. The matrices were parameterized with transmission data that had been collected over the acute phase of the infection [25]. The present study shows that these theoretical models should consider incorporating the decrease in transmission efficiency between the acute and the chronic phase of the infection.

The two strains differed in their spirochete load in the nymphal ticks. We had previously shown that the mean spirochete load in nymphs infected as larvae via systemic transmission during the acute phase of the infection was two-fold higher for strain A10 than for strain A3 [35]. In the present study, we show that this two-fold difference in nymphal spirochete load between strains A10 and A3 is maintained during the chronic phase of the infection (Table 1). We have recently shown in *B. afzelii* that *ospC* strains that maintain a high spirochete load in the nymphal tick are more common in our local population of *I. ricinus* ticks [48]. This observation suggests that the spirochete load is an important life-history trait for *Borrelia* pathogens. Spirochetes have to persist in the flat nymph for a long period of time (~8 months) until the nymph takes its first (and only) blood meal [29, 49]. The spirochete population size inside the tick midgut is likely to be important for maintaining a persistent infection inside the nymphal tick [50–52]. In addition, during the nymphal blood meal, only a small fraction of spirochetes complete the migration from the tick midgut to the tick salivary glands [53–55]. A recent study using genetically tagged strains of *B. burgdorferi* (*s.s*.) found that strains with higher spirochete loads in the nymph have a higher probability of tick-to-host transmission [37]. The ability of strain A10 to establish a high spirochete load inside the nymph may explain why this strain is so common in nature [48].

We recently characterized the community of *B. afzelii ospC* strains in a local population of *I. ricinus* nymphs over a period of 11 years [56]. For the subset of nymphs that were infected with *B. afzelii*, strains carrying *ospC* major group (oMG) A10 (54.4% = 105/193) were almost 12 times more common than strains carrying oMG A3 (4.7% = 9/193) [56]. An experimental infection study found that strain A10 is highly efficient at the three canonical fitness components of any tick-borne pathogen: tick-to-host transmission, host-to-tick transmission (during the acute phase of the infection), and co-feeding transmission [25]. In that study, isolate NE4049 (corresponding to strain A10 in the present study) had the highest R_0_ value among the nine tested isolates of *B. afzelii* [25]. Thus A10 is the most common *B. afzelii ospC* strain in our local population of ticks because it has high fitness (high R_0_ value) [25] and because it maintains a high spirochete load inside the nymphal ticks (which translates into high persistence and high tick-to-host transmission) [48]. Recent studies have shown that oMG A10 is common in other parts of Switzerland [57] and Sweden [58]. Surprisingly, genetic screening of human isolates has never recovered *B. afzelii* oMG A10 from a human patient [59–61]. Future studies should screen human isolates of *B. afzelii* to test whether strains carrying oMG A10 are infectious to humans.

If strain A10 has higher lifetime transmission success than strain A3, what allows strain A3 to persist in nature? One possible explanation is the hypothesis of multiple niche polymorphism (MNP), which suggests that these strains are adapted to different vertebrate reservoir hosts [38, 62–64]. The MNP hypothesis was proposed for *B. burgdorferi* (*s.s*.) in North America where different *ospC* strains appear to be associated with different small mammal hosts [62–64]. In Europe, there are not many studies that have investigated the MNP hypothesis, but one study in France found that an invasive species of chipmunk and the native bank vole carried different strains of *B. afzelii* [65]. In summary, strain A3 could persist in nature if it is adapted to a different vertebrate host than strain A10. Another explanation for the persistence of strain A3 is the competition hypothesis, which suggests that performance in single strain infections is not necessarily predictive of performance in mixed or multiple strain infections. For example, experimental infection studies with rodent malaria and African sleeping sickness have shown that avirulent strains can suppress the density of fast-growing virulent strains [66, 67]. Mixed or multiple strain infections of *Borrelia* pathogens are common in both the vertebrate host [57, 58, 62, 63, 68–70] and the tick vector [48, 56, 57, 62, 71–73]. The mean spirochete load per *Borrelia* strain decreases as strain richness increases in both the vertebrate host and the tick vector, suggesting the presence of competition between *Borrelia* strains [48, 70, 73]. To date, there are only two experimental studies that have investigated mixed *Borrelia* strain infections in the rodent host, but both of these studies were inconclusive with respect to whether co-infection influenced host-to-tick transmission [28, 30]. Thus strain A3 may persist if it is a better competitor than strain A10 in mixed infections in either the vertebrate host or the tick. Future experiments should compare host-to-tick transmission success between mice infected with single strains and mice co-infected with multiple strains.

## Conclusions

The pattern of host-to-tick transmission of *B. afzelii* over the acute and chronic phase of the infection was consistent with the boom-and-bust model of Kurtenbach et al. [7]. The efficiency of systemic transmission decreased between the acute and chronic phase of the infection by 16.1 and 29.3% for strains A3 and A10, respectively. In contrast to studies on North American strains of *B. burgdorferi* (*s.s*.), the present study found no strains with the rapidly cleared phenotype. A3 and A10 were both inhost persistent strains with high host-to-tick transmission over the duration of the infection. Strain A10 had slightly higher host-to-tick transmission and established a consistently higher spirochete load in the nymphal tick than strain A3. Nymphal spirochete load is an important life-history trait for *Borrelia* and may explain why strain A10 is so common in nature.

## Additional file

Additional file 1: Data analysed in the present study. (XLSX 154 kb)

## Abbreviations

GLME: Generalized linear mixed effects
LB: Lyme borreliosis
LME: Linear mixed effects
OspC: Outer surface protein C
PBS: Phosphate-buffered solution
R_0_: Reproductive number
